# The association between locomotive function and incidence of proximal junctional kyphosis following adult spine deformity surgery

**DOI:** 10.1186/s12891-024-08065-x

**Published:** 2024-11-30

**Authors:** Ohsang Kwon, Sanghoon Lee, Haolin Zheng, Dae-Woong Ham, Chungwon Bang, Sang-Min Park, Jin S. Yeom, Ho-Joong Kim

**Affiliations:** 1 Spine Center, Department of Orthopedic Surgery, Seoul National University College of Medicine and Seoul National University Bundang Hospital, 166 Gumiro, Bundang-gu, Sungnam, 463-707 Republic of Korea; 2 Department of Orthopedic Surgery, Chung-Ang University Hospital, Chung-Ang University College of Medicine, Seoul, Republic of Korea

**Keywords:** Functional mobility, Sit-to-stand test, Adult spinal deformity, Proximal junctional kyphosis, Oswestry disability index, Receiver operating characteristics curve

## Abstract

**Background and objectives:**

This retrospective review study aimed to determine whether functional mobility test (FMT) results are related to the incidence of proximal junctional kyphosis (PJK) after surgical correction of adult spinal deformity (ASD).

**Methods:**

A total of 157 patients who underwent reconstructive spinal surgery for ASD between July 2019 and December 2021 were included in this study. Three types of FMTs were performed preoperatively: timed-up-and-go (TUG) test, five times sit-to-stand (STS) test, and alternate step (AS) test. The primary outcome measure was the occurrence of PJK at 1 year after surgery. Oswestry disability index (ODI) and EuroQOL-5-dimension (EQ-5D) scores were surveyed as patient-reported outcome measures of the surgery.

**Results:**

The occurrence of PJK was observed in 41 of the 157 patients (26.1%) at 1 year after surgery. Among the three functional mobility tests, STS test results were significantly higher in the patients who developed PJK. The receiver operating characteristics curve drawn with the STS test had an area under the curve of 0.69 and the optimal cutoff value was suggested as 22 seconds. Multivariate logistic regression analysis identified the STS test along with age and preoperative ODI score as the significant predictors of PJK (*p* = 0.026, 0.005 and 0.001, respectively).

**Conclusions:**

A longer test time on the STS test result was associated with a higher occurrence of postoperative PJK. A cutoff value of 22 s can be suggested. Preoperative surveillance of these patients and providing additional efforts and surgical procedures for the prevention of PJK are anticipated to improve the surgical outcome of ASD correction.

## Introduction

With the current trend of increase in the senile population, the demand for proper treatment of adult spinal deformity (ASD) has also been increasing. Although surgical treatment of ASD is believed to be associated with a higher likelihood of clinical improvement compared to conservative treatment [1], postoperative complications such as proximal junctional kyphosis (PJK) are still a problem to be resolved [2–5].

Many studies have revealed numerous risk factors that might lead to the occurrence of PJK [4, 6–8], and various methods have been proposed to prevent PJK and thus, proximal junctional failure (PJF) [9–11]. However, these attempts could not effectively decrease the incidence of PJK [12]. Hence, efforts to elucidate the powerful predictors of PJK are still in progress, although the pathophysiology of PJK is multifactorial.

Risk factors of PJK that have been proposed include older age, fusion down to the sacrum or pelvis, larger thoracic kyphosis, low bone mineral density (BMD), and a preoperative sagittal vertical axis (SVA) difference greater than 50 mm [13]. The focus of studies has been on the analysis of two-dimensional static images or descriptive factors of the patient population. On the other hand, the disabilities and pain that ASD patients manifest are grounded in the dynamic aspects of daily performance. Recent studies have highlighted the physical function of patients via dynamic motion analysis or balance tests to understand the disease itself and to relate with the beneficial or adverse outcomes of surgical correction [14, 15].

Therefore, this study hypothesized that the measurement of the physical function of patients can predict the postoperative development of PJK. We used three validated functional mobility tests (FMTs) to assess the locomotive function associated with frailty: the Timed-Up-and-Go (TUG) test, the five times Sit-to-Stand (STS) test, and the Alternate Step (AS) test [16–18]. This study aimed to examine the relationship between locomotive function and the development of PJK.

## Materials and methods

### Study design and patients

This retrospective review study was approved by the institutional review board of our hospital (B-2104-678-104). Informed consent was obtained regarding the surgical procedure, its potential outcomes, and possible complications and that imaging data, surgical outcomes, and medical records may be used for retrospective research purposes. A total of 157 consecutive patients who were scheduled to undergo reconstructive spinal surgery for ASD between July 2019 and December 2021 were included in the study. The inclusion criteria for the ASD group were as follows: (1) age > 50 years; (2) diagnosis of ASD with sagittal imbalance and treatment plan for corrective surgery, defined as SVA > 5 cm, pelvic tilt (PT) > 20°, or pelvic incidence (PI) – lumbar lordosis (LL) > 20° on lateral radiographs in standing position; and (3) subjective disability due to stooping posture. The exclusion criteria were as follows: (1) presence of any other spinal disease such as thoracic and/or cervical myelopathy; (2) severe pain in the hip, knee, or ankle joints impeding walking; (3) peripheral vascular disease; (4) any syndromic, neuromuscular disease; (5) coronal imbalance of Cobb angle > 25°; (6) having undergone revision surgery due to complications such as rod breakage; and (7) any serious uncontrolled medical comorbidity such as sepsis or malignancy that would cause disability or worsen the general medical condition.

### Functional mobility measurements

We performed three different types of functional mobility tests. The tests comprised of the TUG test, STS test, and AS test. The TUG test measured the time it took for the subject to stand up from a chair with no armrest, walk a distance of 3 m, turn, walk back to the chair, and sit down. It has been developed as a clinical measure of balance in elderly people [19]. The STS test measured the time it took for the subject to stand up from a chair with no armrest, sitting back down, and repeating it five times [17]. The AS test measured the time it took for the subject to climb a single foot-high step with right and left foot, alternatively, for eight times in total [20]. Handgrip strength (HGS) was measured for both hands using a hand dynamometer (GRIP-D5101, Takei, Niigata, Japan). The patients were asked to sit in a comfortable position with their elbows extended to the side and squeeze the dynamometer with maximum strength. Measurements were performed twice for both hands with a minute break in between [21]. The best performance of the trials was recorded and entered into the analysis, regardless of the side and dominance of the hand used.

### Surgical procedures

All surgeries were conducted by the lead author using consistent techniques [22, 23]. Patients were positioned prone on a Jackson spine table to achieve maximal LL, and simple radiographs were taken. The PI – LL was calculated to determine the desired correction angle and whether a 3-column osteotomy or posterior column osteotomy was needed to correct sagittal imbalance. The fusion level was carefully determined by evaluating both sagittal and coronal plane deformities with most patients receiving fusion down to the sacrum and iliac screws to support the long construct and prevent early degeneration of the L5/S1 disc. The upper instrumented vertebra (UIV) was T10 in all patients. Methods including interbody fusion with a cage and bone graft at the lower lumbar levels, rod cantilevering, and compression between screws were employed to help correct the deformity.

### Surgical outcome measurements

Surgical outcome measurements were generated using radiographic measurements and patient-reported outcome (PRO) measures. For radiographic assessment, spinopelvic parameters, including the SVA, sacral slope, PT, PI, and LL, were measured using biplanar stereo radiographic full-body imaging (EOS imaging, Paris, France). PJK was defined by the presence of two criteria: (1) a proximal junctional sagittal Cobb angle (PJA) of > 10° and (2) a postoperative PJA of at least 10° greater than the preoperative measurement [4]. ‘Acute PJK’ refers to PJK occurring within the first year postoperatively. PRO measures, including the Oswestry Disability Index (ODI) and EuroQOL (EQ-5D) were used to assess surgical outcomes [24, 25]. The ODI is a self-administered questionnaire that measures “back-specific function” on a 10-item scale, with six response categories each [25]. The EQ-5D is a 5-dimensional health state classification; the five dimensions are mobility, self-care, usual activities, pain/discomfort, and anxiety/depression [24, 26]. An EQ-5D “health state” is defined by selecting one level from each dimension. The EQ-5D preference-based measure can be regarded as a continuous outcome scored on a 0 to 1.00 scale, with 1.00 indicating “full health” and 0 representing death. These data were collected preoperatively and reassessed at 3 and 6 months after surgery. Patients who experienced PJK events before the PROs were conducted were excluded.

### Statistical analysis

Continuous variables were expressed as means with standard deviations for parametric data, or medians with interquartile ranges (IQR) for non-parametric data. Preoperative PROs, radiological parameters, and demographic data were compared between the two groups using independent *t*-tests or the Mann-Whitney U test. The incidence of PJK at 1 year after surgery, the primary outcome measure of our study, and the functional mobility test results were analyzed. The area under the curve (AUC) and the optimal cutoff values were calculated from the receiver operating characteristic (ROC) curve to evaluate the ability of the functional mobility test to predict the occurrence of PJK. Variables that were significantly associated with the development of acute PJK in the univariate analysis (*p* < 0.10) were then entered into the multivariate logistic regression model after excluding the possibility of collinearity. The TUG, STS, and AS test values were analyzed by categorizing them based on the cutoff value from the ROC curve. Finally, a multivariate logistic regression model was used to identify risk factors and calculate the odds ratio (OR) for acute PJK. All statistical analyses were performed using SPSS 27.0 (SPSS, Inc., Chicago, IL, USA). Statistical significance was set at *p* < 0.05.

## Results

### Descriptive analysis

A total of 157 patients with a mean age of 71.5 ± 7.3 years were enrolled in the study. Baseline demographic parameters and functional mobility test results were compared with the occurrence of PJK (Table 1). PJK occurred in 41 of 157 patients (26.1%) at 1 year after surgery. Age and HGS exhibited significant differences according to the occurrence of PJK (*p* = 0.001 and 0.001, respectively). Among the FMTs, the PJK + group needed a longer time to complete the STS test than the PJK– group with statistical significance (23.9 ± 9.4 vs. 20.2 ± 9.3 s, *p* = 0.028). There was no statistically significant difference between the PJK + and PJK- groups for the TUG test (18.2 ± 6.9 vs. 17.0 ± 6.9 s, *p* = 0.354) or the AS test (16.4 ± 8.0 vs. 16.1 ± 7.4 s, *p* = 0.871). Sex, body mass index, and BMD were not significantly different between the PJK + and PJK– groups. Preoperative ODI scores were better in the PJK + group but the EQ-5D scores of PJK + group after surgery were worse (Table 1).

**Table 1:** Comparative analysis of the patient characteristics and functional mobility test by the occurrence of proximal junctional kyphosis

	PJK +	PJK –	Total	*p*-value
No. (%)	41 (26.1%)	116 (73.9%)	157	
Age	74.7 ± 5.0	70.4 ± 7.7	71.5 ± 7.3	**0.001**
Sex, female (%)	37 (90.2%)	101 (87.1%)	138 (87.9%)	0.592
BMI	26.9 ± 3.9	26.2 ± 3.9	26.4 ± 3.9	0.335
HGS (kg)	13.5 ± 4.2	19.4 ± 17.3	17.8 ± 15.2	**0.001**
BMD (g/cm^2^)	0.608 ± 0.10	0.642 ± 0.11	0.633 ± 0.11	0.097
ODI				
Preop	37 (24–50)	46 (34–54)	44 (33–54)	**0.038**
Postop	42 (24–56)	34 (20–50)	36 (21–50)	0.185
EQ-5D				
Preop	0.296 (0.081–0.553)	0.196 (0.081–0.446)	0.196 (0.081–0.482)	0.720
Postop	0.410 (0.143–0.553)	0.410 (0.081–0.410)	0.410 (0.109–0.553)	**0.040**
Functional mobility test				
TUG	18.2 ± 6.9	17.0 ± 6.9	17.3 ± 6.9	0.354
STS	23.9 ± 9.4	20.2 ± 9.3	21.2 ± 9.4	**0.028**
AS	16.4 ± 8.0	16.1 ± 7.4	16.2 ± 7.5	0.871

PJK, proximal junctional kyphosis; BMI, body mass index; HGS, hand grip strength; BMD, bone mineral density; ODI, Oswestry disability index; EQ-5D, EuroQOL 5- dimension; TUG, timed up-and-go; STS, sit-to-stand; AS, alternate step

*p*-value < 0.05 are shown in **bold**

### Radiological outcome analysis

All preoperative spinopelvic radiologic parameters showed no statistically significant differences between the PJK + and PJK– groups. However, patients of the PJK + group displayed a larger PT and LL values after surgery (Table 2).

**Table 2:** Comparative analysis of radiologic parameters by the the occurrence of proximal junctional kyphosis

	PJK +	PJK –	Total	*p*-value
Preop				
SS	23.8 ± 10.9	24.8 ± 14.4	24.5 ± 13.6	0.68
PT	29.2 ± 9.1	28.1 ± 11.6	28.4 ± 11.0	0.56
PI	53.0 ± 11.2	52.7 ± 13.5	52.8 ± 12.9	0.91
LL	5.2 ± 22.8	6.9 ± 23.2	6.5 ± 23.0	0.68
PI–LL	47.8 ± 24.6	46.1 ± 23.0	46.5 ± 23.4	0.68
SVA	171.1 ± 86.2	177.1 ± 80.7	175.6 ± 81.9	0.70
PJA	2.1 ± 5.2	2.7 ± 5.9	2.5 ± 9.5	0.58
Postop				
SS	24.7 ± 9.3	26.1 ± 9.6	25.7 ± 9.5	0.45
PT	28.8 ± 8.9	25.4 ± 8.8	26.3 ± 8.9	**0.04**
PI	53.6 ± 11.0	51.5 ± 12.0	52.0 ± 11.7	0.34
LL	39.8 ± 9.4	35.5 ± 10.7	36.6 ± 10.5	**0.03**
PI–LL	13.8 ± 11.3	15.9 ± 10.9	15.4 ± 11.0	0.30
SVA	66.2 ± 41.2	59.4 ± 42.4	61.2 ± 42.2	0.39

SS, sacral slope; PT, pelvic tilt; PI, pelvic incidence; LL, lumbar lordosis; PI–LL, pelvic incidence minus lumbar lordosis; SVA, sagittal vertical axis

*p*-value < 0.05 are shown in **bold**

### Predictive validity and the cutoff value of functional mobility test for the PJK

**Fig. 1:**
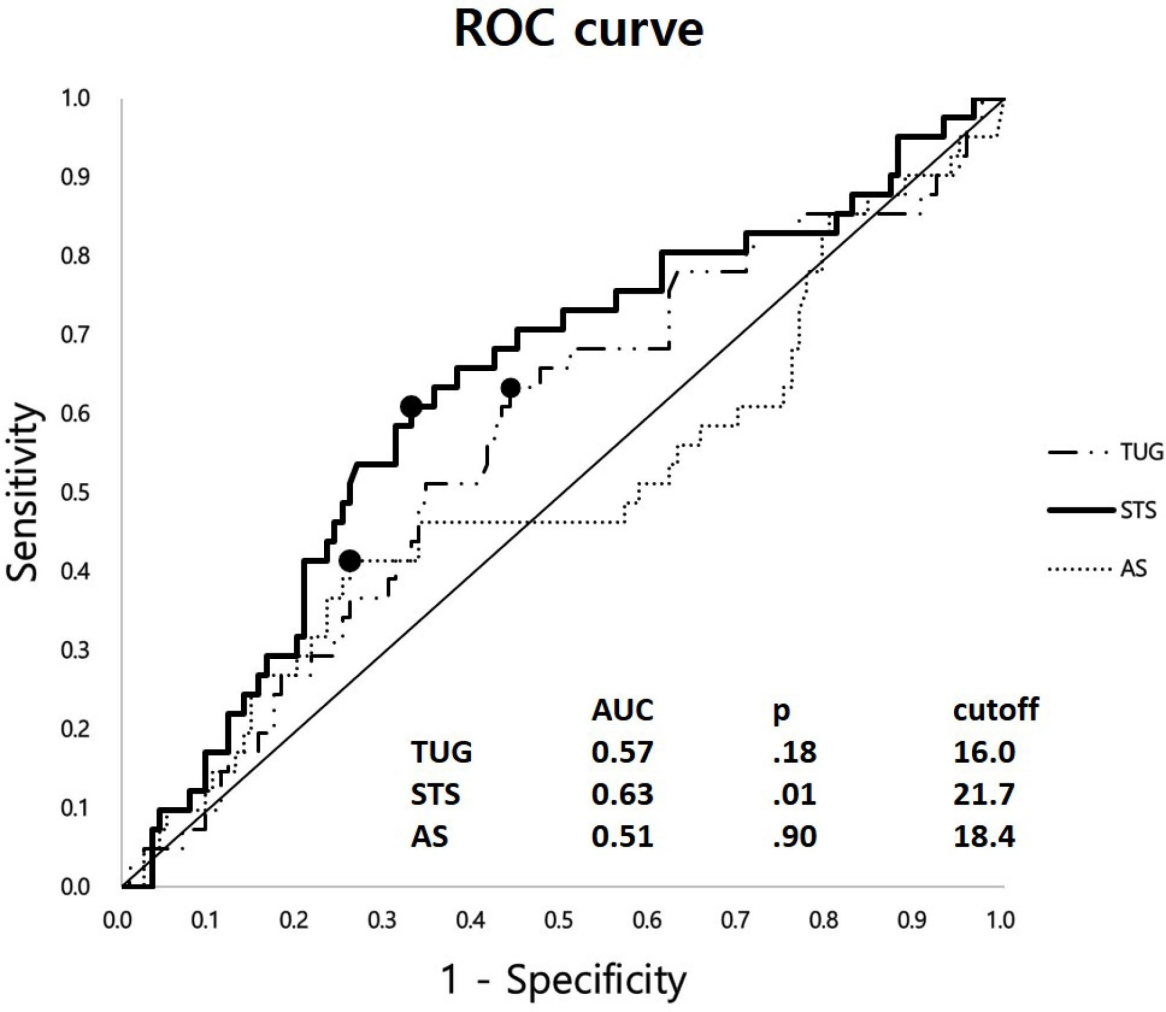
A receiver operating characteristics (ROC) curve of the three functional mobility tests (FMT) for prediction of the occurrence of proximal junctional kyphosis (PJK). The area under the curve (AUC), *p*-value, and the optimal cutoff values are suggested in the table

The AUC of the ROC curves were 0.63, 0.57, and 0.51 for the STS, TUG, and AS tests (*p* = 0.01, 0.18, and 0.90, respectively). The suggested optimal cutoff values were 21.7, 16.0, and 18.4 for the STS, TUG, and AS tests (Fig. 1).

### Risk factors for the development of acute PJK

Univariate analysis identified that patients’ age, BMD, HGS, preoperative ODI, postoperative PT, postoperative LL, TUG test, and STS test could be multiple potential risk factors for developing acute PJK (Table 3). The TUG and STS test results were dichotomized using the previously suggested cutoff values. Multivariate logistic regression analysis identified age, preoperative ODI, and the STS test as significant risk factors for acute PJK (*p* = 0.005, 0.001, 0.026, respectively) (Table 3).

**Table 3:** Univariate & multivariate logistic regression analysis for prediction of the proximal junctional kyphosis

	Univariate analysis			Multivariate analysis		
	β	Odds ratio (95% CI)	*p*-value	β	Odds ratio (95% CI)	*p*-value
Age	0.12	1.13 (1.0–1.2)	**0.001**	0.13	1.1 (1.0–1.2)	**0.005**
Sex (female)	0.32	1.37 (0.4–4.4)	0.593			
BMD (g/cm^2^)	-2.9	0.05 (0.0–1.8)	**0.099**	-2.50	0.08 (0.00–8.34)	0.289
HGS	-0.09	0.91 (0.8–0.9)	**0.006**	-0.05	0.95 (0.87–1.04)	0.251
Preop ODI	-0.03	0.97 (0.95–0.99)	**0.019**	-0.05	0.95 (0.93–0.98)	**0.001**
Postoperative radiologic parameters						
SS	-0.02	0.99 (0.95–1.02)	0.449			
PT	0.05	1.05 (1.00–1.09)	**0.043**	0.03	1.03 (0.98–1.08)	0.324
PI	0.02	1.02 (0.98–1.05)	0.335			
LL	0.04	1.04 (1.00–1.08)	**0.032**	0.04	1.04 (0.99–1.09)	0.068
PI-LL	-0.02	0.98 (0.95–1.02)	0.297			
SVA	0.00	1.00 (0.99–1.01)	0.389			
TUG test*	0.76	2.1 (1.0–4.5)	**0.044**	-0.07	0.93 (0.32–2.72)	0.905
STS test*	1.06	2.9 (1.4–6.0)	**0.004**	0.99	2.70 (1.13–6.47)	**0.026**
AS test*	0.61	1.8 (0.8–3.9)	0.114			

BMD, bone mineral density; HGS, hand grip strength; SS, sacral slope; PT, pelvic tilt; PI, pelvic incidence; LL, lumbar lordosis; PI–LL, pelvic incidence minus lumbar lordosis; SVA, sagittal vertical axis; TUG, timed-up-and-go; STS, sit-to-stand; AS, alternate step

*P*-values < 0.10 for univariate analysis and < 0.05 for multivariate analysis are shown in **bold**.

*Functional mobility test results were converted into categorical variables according to the respective cutoff values suggested from the receiver operating characteristics curve (cutoffs were 16.0, 21.7, 18.4 s for the TUG, STS, and AS test respectively)

### Subgroup analysis by dichotomization with the cutoff of sit-to-stand test

Patients with good performance of STS test were compared with those with poor performance by the cutoff of 21.7 s (Table 4). PJK incidence was significantly higher (38.7% vs. 17.9%, *p* = 0.004) in those with poor performance of the test. This group of poor performance was older with statistical significance (*p* = 0.004). As of the PRO measures, postoperative ODI and EQ-5D scores were better in the group with better STS performance but the difference was statistically insignificant. The radiographic parameters, including postoperative PI–LL mismatch and SVA were in similar range between the two groups, as with the surgical parameters including fusion length, iliac screw insertion, and 3-column osteotomy (Table 4).

**Table 4:** Subgroup analysis of patients by dichotomization with the 21.7 s cutoff of sit-to-stand test

	Good STS performance (< 21.7 s)	Poor STS performance (> 21.7 s)	*p*-value
No. (%)	95 (60.5%)	62 (39.5%)	
PJK incidence (n, %)	17 (17.9%)	24 (38.7%)	**0.004**
Age	70.2 ± 8.0	73.6 ± 5.4	**0.004**
Sex, female (%)	80 (84.2%)	58 (93.5%)	0.079
BMI	26.2 ± 3.7	26.8 ± 4.2	0.312
HGS (kg)	17.6 ± 8.2	18.2 ± 22.1	0.804
BMD (g/cm^2^)	0.643 ± 0.1	0.619 ± 0.1	0.176
ODI			
Preop	42 (31–52)	46 (34–54)	0.444
Postop	33 (22–50)	38 (20–56)	0.418
EQ-5D			
Preop	0.211 (0.081–0.482)	0.196 (0.081–0.553)	0.716
Postop	0.410 (0.143–0.553)	0.410 (0.103–0.553)	0.247
PI–LL			
Preop	46.3 ± 23.5	47.0 ± 23.3	0.846
Postop	15.7 ± 10.8	14.8 ± 11.4	0.600
SVA			
Preop	172.2 ± 83.9	180.8 ± 79.1	0.525
Postop	59.8 ± 40.6	63.4 ± 45.0	0.619
PJA			
Preop	1.95 ± 6.03	3.37 ± 5.04	0.126
Postop	10.11 ± 8.70	13.93 ± 10.46	**0.016**
Fusion length	8.0 ± 0.7	8.0 ± 0.3	0.729
Iliac screw insertion	70 (86.4%)	41 (95.3%)	0.216
3-column osteotomy	25 (30.9%)	12 (27.9%)	0.732

STS, sit-to-stand test; PJK, proximal junctional kyphosis; BMI, body mass index; HGS, hand grip strength; BMD, bone mineral density; ODI, Oswestry disability index; EQ-5D, EuroQOL 5- dimension; PI–LL, pelvic incidence and lumbar lordosis mismatch; SVA, sagittal vertical axis; PJA, proximal junctional sagittal Cobb angle

*p*-value < 0.05 are shown in **bold**

## Discussion

Despite the growing body of research on PJK, there remains a gap regarding the potential association between the locomotive function of patients and the occurrence of PJK. This study shows that poor performance of the STS test is associated with a higher incidence of PJK. A cutoff of 22 s would be realistic in the clinical settings. The odds ratio of developing PJK for the patients who took longer time than cutoff to complete the STS test was 2.70 (95% confidence interval: 1.13–6.47) (Table 3).

Conventionally suggested risk factors of PJK include older age, long spinal fusion construct, larger correction of the deformity, and low BMD [13]. However, interest has been growing in the dynamic and functional aspects of patients with ASD recently. Severijns et al. reported from data using motion analysis that ASD patients showed altered lower limb gait patterns to compensate for trunk tilt and pelvic anteversion during walking, which correlates with an increased risk of falls [27]. Godzik et al. recently reported a prospective pilot study using static and dynamic posturography, demonstrating that ASD patients had impaired postural stability compared to the normal population, which might elevate the risk of falls [28]. Furthermore, Kim et al. demonstrated increased risk of falls in patients with spinal stenosis using FMT, representing the significance of the ability to maintain postural stability [29]. Based on this background, we investigated whether FMT can be used as a measurement tool to evaluate the dynamic stability of ASD patients.

We suggest that patients with a higher probability of developing PJK can be successfully screened using 22 s as cutoff value of the STS test. In particular, the STS test showed superiority in predicting the PJK incidence over other FMTs, the TUG and AS tests. This might be explained by the nature of the STS activity and the pathophysiology of the development of PJK. The STS test is a repetition of getting up and sitting down onto a chair. Getting up and sitting down onto a chair is a form of an anti-gravitational activity. Therefore, decreased core muscles, impaired locomotive function, and compromised balancing ability result in poor performance for these tests, ultimately leading to increased stress load and impact at the proximal junction of the long fusion construct. Understandably, this will lead to wedge compression of the UIV or UIV + 1, which is the common pathophysiology of PJK. Evaluation of the patients’ muscle quality by direct measurement of body composition or surveying known associated factors of muscle function and comparing them with the STS test results would have been of help in understanding the correlation. We have included factors like HGS, BMI, and BMD in our analysis but alcohol consumption, smoking, or comorbidities are also known to have impact on the STS test results [30]. We also excluded the patients diagnosed with syndromic or neuromuscular diseases such as Parkinson’s disease or brain diseases that might affect the functional mobility. Diebo et al. [31] reported that older patients took longer to complete the Dubousset functional test (DFT), which evaluates physical function and balance. DFT is similar to the FMT of the present study in that it includes sitting and getting up motions. Furthermore, the tests included in FMT were validated by previous studies [29, 32], in which the patients who took a longer time to complete these tests had a higher risk of falls. In addition, Staartjes et al. proposed a severity stratification for objective functional impairment (OFI) in patients with degenerative lumbar spine disease based on STS test times, categorizing times greater than 22.0 s as indicative of severe OFI [33]. Similarly, Klukowska et al. demonstrated that the five-repetition sit-to-stand test could predict falls in older adults using a cutoff of 23.8 s [34]. This aligns with our cutoff of 22 s for predicting PJK, further emphasizing the role of functional impairment in postoperative outcomes. In this context, it is a noteworthy point of the present study, that the results of functional mobility tests that measure patients’ locomotive function are correlated with the occurrence of PJK. While it may be debatable whether a small difference in the STS test is a significant contributing factor to the occurrence of PJK, it can be considered as one potential risk factor in the complex interplay of multiple mechanisms leading to PJK. Therefore, the value of the STS test lies in assisting the surgeon to quickly assess and comprehend the functional ability of the patients with ASD as a simple clinical tool.

One of the notable findings in our study is the significant difference in age between patients with longer STS test times and those with shorter test times. Patients with poor STS test performance (greater than 22 s) were on average 3.4 years older than those with better performance (73.6 vs. 70.2 years). Given that age was also identified as a significant risk factor for PJK in the multivariate analysis, it is possible that the age difference contributed to both the variation in STS test performance and the higher incidence of PJK in older patients. It is likely that age and STS test time are interrelated factors, both contributing to the higher incidence of PJK. While the STS test serves as a valuable tool for assessing functional mobility, it is important to consider that the test results may be influenced by patient age. This potential confounding factor should be considered when interpreting the findings of this study. Further research could focus on exploring the interaction between age and FMTs in predicting PJK, and on evaluating whether interventions aimed at improving mobility in older patients could reduce the risk of PJK.

It is widely known that frailty is a significant factor in the development of PJK [13, 35]. HGS is a representative frailty-related factor, and previous studies have reported that patients with weak HGS show poor outcomes after surgery [22]. In our data, the PJK + group showed weaker HGS (13.5 ± 4.2 vs. 19.4 ± 17.3, *p* = 0.001). Likewise, previous reports demonstrated that tests including FMT could be routinely used for medical screening tests in frail, elderly population [19, 36]. In this aspect, FMT can be a non-invasive method for screening patients’ frailty easily and objectively, allowing surgeons to identify among ASD patients of who is expected to exhibit worse outcomes after surgery.

This study has several limitations. First, the follow-up period was 1 year after surgery. Although this is a rather short follow-up period, majority of the PJK occurs within this acute phase of the postoperative period, reported as 66% within the first 3 months and 80% within 18 months after surgery [37]. Nevertheless, a longer follow-up study would be necessary to confirm the relationship between the STS test and the PJK. Second, the study was designed to determine whether functional mobility tests can predict the incidence of PJK. Since this possibility has now been proven, more mechanistic studies such as motion analysis of the STS activity rather than simple walking, or linking other factors representing muscle functional status, could be incorporated to examine the mechanism of PJK development and ultimately to prevent troublesome complications. Third, we chose to include only patients above the age of 50. However, patients younger than 50 could meet the diagnostic requirements for ASD and may benefit from surgical correction, and mobility test outcomes may differ for these younger patients. Fourth, although the FMT test is known to have different cutoffs for the risk of falls according to age, present study did not consider the association between age and the FMT results. However, the fact that present study included relatively homogenous elderly patients could minimize bias. Lastly, an AUC of 0.63 in the STS test indicates neither high sensitivity nor specificity, but it is considered acceptable.

## Conclusion

Poor performance of the STS test, especially over 22 s leads to higher risk of PJK occurrence. Surveillance of these patients and providing additional procedures for the prevention of PJK are anticipated to improve the surgical outcome of ASD correction.

## Data Availability

The datasets used and/or analysed during the current study are available from the corresponding author on reasonable request.

## Abbreviations

FMT: Functional mobility test
PJK: Proximal junctional kyphosis
ASD: Adult spinal deformity
TUG: Timed-up-and-go
STS: Sit-to-stand
AS: Alternative step
ODI: Oswestry disability index
EQ-5D: EuroQOL-5-dimension
PJF: Proximal junctional failure
BMD: Bone mineral density
SVA: Sagittal vertical axis
PT: Pelvic tilt
PI: Pelvic incidence
LL: Lumbar lordosis
HGS: Handgrip strength
UIV: Upper instrumented vertebra
PRO: Patient-reported outcome
PJA: Proximal junctional sagittal Cobb angle
AUC: Area under the curve
ROC: Receiver operating characteristic
OR: Odds ratio
